# Appraisal on the wound healing potential of *Melaleuca alternifolia* and *Rosmarinus officinalis* L. essential oil-loaded chitosan topical preparations

**DOI:** 10.1371/journal.pone.0219561

**Published:** 2019-09-16

**Authors:** Rola M. Labib, Iriny M. Ayoub, Haidy E. Michel, Mina Mehanny, Verena Kamil, Meryl Hany, Mirette Magdy, Aya Moataz, Boula Maged, Ahmed Mohamed

**Affiliations:** 1 Department of Pharmacognosy, Faculty of Pharmacy, Ain Shams University, Cairo, Egypt; 2 Department of Pharmcology and Toxicology, Faculty of Pharmacy, Ain Shams University, Cairo, Egypt; 3 Department of Pharmaceutics and Industrial Pharmacy, Faculty of Pharmacy, Ain Shams University, Cairo, Egypt; 4 Helmholtz Institute for Pharmaceutical Research Saarland (HIPS), Helmholtz Center for Infection Research (HZI), Saarland University, Saarbrücken, Germany; 5 Drug Design Program, Faculty of Pharmacy, Ain Shams University, Cairo, Egypt; King Abdulaziz University, SAUDI ARABIA

## Abstract

The present study investigates the wound healing potential of three chitosan-based topical preparations loaded with either tea tree essential oil, rosemary essential oil or a mixture of both oils *in vivo*. Essential oils of *M*. *alternifolia* and *R*. *officinalis* were analyzed using GC/MS. Essential oil-loaded chitosan topical preparations were formulated. Wound healing potential was evaluated *in vivo* using an excision wound model in rats. GC/MS analysis of *M*. *alternifolia* and *R*. *officinalis* essential oils revealed richness in oxygenated monoterpenes, representing 51.06% and 69.61% of the total oil composition, respectively. Topical application of chitosan-based formulation loaded with a mixture of tea tree and rosemary oils resulted in a significant increase in wound contraction percentage compared to either group treated with individual essential oils and the untreated group. Histopathological examination revealed that topical application of tea tree and rosemary oil combination demonstrated complete re-epithelialization associated with activated hair follicles. The high percentage of oxygenated monoterpenes in both essential oils play an important role in the antioxidant and wound healing potential observed herein. Incorporation of tea tree and rosemary essential oils in chitosan-based preparations in appropriate combination could efficiently promote different stages of wound healing.

## Introduction

Traditional remedies, especially plant-based formulations, promote wound healing in a striking way, since they influence single or multiple stages during the wound healing cascade. This cascade comprises several systematic physiological events aiming to repair the damaged tissue either completely or at least partially [1]. The process could be categorized into 3 main phases; the inflammatory phase (re-establishment of homeostasis and local inflammation), the proliferative phase (granulation, contraction of tissue and beginning of epithelialization) and ultimately the remodeling phase, which decides the strength and appearance of tissue after healing [2].

Essential oils, isolated from various plant sources, are commonly utilized in first aid treatment of wounds, burns or abscesses. Recent studies shed the light on their unique antimicrobial, pesticidal, wound-healing promoting and antioxidant properties, owing to their pharmacologically active compounds, e.g. borneol, camphor, terpinen-4-ol, eucalyptol and many other compounds [3].

Tea tree oil is the essential oil derived from the Australian native plant *Melaleuca alternifolia* (Maiden &Betche) Cheel belonging to family Myrtaceae which grows naturally in Northern New South Wales, Australia [4]. The anti-bacterial and antiseptic properties of tea tree oil are legendary and have been used for centuries. The local Aborigine tribes used to crush the leaves, make poultices and bound them to the skin to heal cuts, burns, bites, and stings [5]. It was officially identified as an antiseptic in 1923 by Dr. Arthur Penfold who reported that tea tree oil was11 times stronger in activity than phenol, the standard antiseptic at that time. This “magic healing oil” was supplied to the Australian army during World War II as it was the best possible available treatment against infection from cuts, wounds and bites [5–9].

Rosemary essential oil is obtained from the aromatic herb *Rosmarinus officinalis* L. (Lamiaceae), a woody perennial herb, native to the Mediterranean region [10]. In folk medicine, *R*. *officinalis* has been used to treat headaches, poor circulation, epilepsy, a mild analgesic and anti-inflammatory [11]. Rosemary essential oil was reported to possess strong antioxidant and antimicrobial properties as well as wound healing activity [10, 12]. Moreover, topical application of tea tree and rosemary essential oils has been well documented with satisfactory safety and efficacy [6–15]

Chitosan [16] confers a plethora of privileges owing to its biocompatibility, biodegradability, film-forming, penetration-enhancement, wound healing and anti-microbial characteristics [16], Several studies provided adequate evidence for the synergistic effects of CS with essential oils to prevent infection and enhance wound healing [17]. The positively charged surface groups of CS, which can interact with the bacterial negatively charged groups, account for its antimicrobial effect [18], while essential oil bactericidal effect can be ascribed to its phenolic and aldehydic content [19]. Polyvinyl alcohol (PVA) shows very promising film-forming and wound healing qualities, especially when formulated with CS, in addition to its inherent emulsifying and viscosity modifying properties which stabilizes essential oils within CS films [20]. The objective of this research was to elucidate the wound healing ability of CS films loaded with essential oils, rosemary oil or tea tree oil, either separately or in combination.

## Materials and methods

### Chemicals and reagents

Rosemary and tea tree essential oils were purchased from Harraz - Agricultural Seeds, Spices &Medical Plants Co., Bab El Khalk, Cairo, Egypt. Nolaver cream was obtained from Parkville pharmaceuticals, Egypt (Batch no. 001/15). Highly viscous CS from crab shells, PVA (Mowiol 4–88) molecular weight ∼31,000 and glacial acetic acid were purchased from Sigma Aldrich, St. Louis, USA. Glycerol was purchased from El-Nasr company, Cairo, Egypt. Kits of malondialdehyde and reduced glutathione were purchased from Biodiagnostic, Egypt.

### GC/MS analysis of essential oils

Essential oils were analyzed using a Shimadzu QP2010 gas chromatograph coupled to a quadrupole mass spectrometer (Shimadzu Corporation, Kyoto, Japan). Essential oil components were separated on Rtx-5MS column (30 m x 0.25 mm inner diameter x 0.25 μm film thickness), Restek, USA. The gas chromatograph was operated under the following conditions: Injector temperature, 250°C; oven temperature 45°C for 2 min, then programmed to 300°C at a rate of 5°C/min, and finally kept constant at 300°C for 5 min. Injections were made in split mode (1:15) with injection volume 1 μl. Helium was used as a carrier gas at 1.41 mL/min. The interface temperature was set to 280°C. Shimadzu quadrupole mass spectrometer was operated at 70 eV in the electron ionization mode and ion source temperature of 200°C, scanning from 35 to 500amu.

Essential oil components were identified by comparison of their retention indices (RI) relative to standard *n*-alkanes (C8-C28); mass spectral data (MS) to NIST and WILEY mass spectral library database; (similarity index > 90%); and literature [21–23].

### Film preparation

Essential oil-loaded CS film was formulated, adopting the method developed by Wang et al. with slight modifications [24]. Briefly, 2% (w/w) viscous CS solution was prepared in 1% (v/v) acetic acid in deionized water (DI H_2_O), with the aid of overnight magnetic stirring. 2% w/v PVA solution was prepared in DI H_2_O *via* magnetic stirring with slight heating. Insoluble particles were removed by filtration through a filter cloth. Solutions of CS and PVA were blended together with glycerol in the ratio of 3:1:1 respectively, and agitated for 1 h until a homogenous, viscous film-forming solution was obtained. Afterwards, selected essential oils were added drop wise onto the viscous solution, with continuous stirring, in order to reach 10% v/v oil concentration within the formulation, and stirred for 1h.Dose selection for essential oils was performed as previously reported [24], where 10% v/v gave better wound-healing outcomes in comparison with other lower concentrations and demonstrated favorable anti-bacterial and wound-healing effects.

Then, essential oil-impregnated film-forming solutions were ultrasonically treated for 10 min to expel air bubbles. An aliquot of 15g film-forming solution was cast onto Plexiglas plates (8.0, 8.0 cm) and allowed to dry for 48h at 25 ± 2°C and 50 ± 2% relative humidity, and finally the films were peeled-off from them.

### *In vivo* wound healing experiment

#### Animals

Thirty-six healthy adult male Sprague Dawley rats weighing 160-180g were purchased from the Nile Company, El Amyria, Cairo, Egypt. Plastic cages were used for animal housing at a constant temperature (21 ± 2°C), with alternating 12h light/dark cycle. Animal chow and water were provided *ad libitum*. All animals were acclimatized to laboratory conditions for one week before the experiment. Efforts were made to ensure minimum animal suffering and to use the smallest possible number of animals. All animal treatments strictly adhered to institutional and ethical guidelines of the care and use of laboratory animals and complied with the National Institutes of Health guide for the care and use of Laboratory animals (NIH Publications No. 8023, revised 1978). The experimental protocol was approved by Ain Shams University, Faculty of Pharmacy Review Committee for the use of animal subjects; permit number: 45

#### Wound creation, animal grouping and drug administration

The anterior dorsal side of each rat was shaved using sterile surgical blade and a two cm^2^ full thickness excision wound was created by removing a patch of skin under ketamine hemisulfate (100 mg/kg, i.p.) anesthesia [25]. A total of 36 animals (160–180 g) were randomly divided into six groups as follows:

Group I:negative control (wound)

Group II: positive control (Nolaver, marketed product, well-known for its wound healing properties)

Group III: plain (chitosan)

Group IV: tea tree oil in chitosan

Group V: rosemary oil in chitosan

Group VI: tea tree and rosemary essential oils mixture (1:1) in chitosan

Nolaver (0.5 g), plain ointment (chitosan) and each test ointment were topically applied on the wounded site of the respective groups once a day throughout the experiment. At the end of the experimental period (14 days), rats were euthanized by decapitation following anesthesia *via* ketamine hemisulfate (100mg/kg, i.p.) and the wound granulation tissues formed were removed and used for further analysis.

#### Wound contraction measurements

The percentage of wound contraction was determined as previously described [26]. During wound area measurement, animals were placed on bench top with the wound facing upwards. A firm but flexible transparent polythene rectangular (3 × 3 cm^2^) sheet was held just over the wound and its margins were marked with a fine tip permanent marker on sheet and the rat was returned back to its cage. The area (mm^2^) within the boundaries of each tracing was determined planimetrically where a standard quality card paper was used to convert the area of the wound on the transparent sheet into the weight of the card paper with the same area. The weight of the card paper/unit area was already known, therefore, the weight of each card paper for a particular wound was easily estimated. Wound area was measured on day 0 and on days 7 and 14 post-wounding. The percentage of wound contraction was used to express the results of wound measurements and was calculated by Wilson's formula as follows[27]:

$$\%\mathrm{wound}\;\mathrm{contraction}=\frac{\mathrm{Day}\;0\;\mathrm{wound}\;\mathrm{area}‐\mathrm{wound}\;\mathrm{area}\;\mathrm{on}\;a\;\mathrm{particular}\;\mathrm{day}}{\mathrm{Day}\;0\;\mathrm{wound}\;\mathrm{area}}\times 100$$

#### Histopathology

Control and treated animals were sacrificed at the end of experimental period and tissues from the wound site of each animal were removed. Then, 10% formalin was used for sample fixation, followed by dehydration using alcohol, clearance in xylene and embedding in paraffinwax. Serial sections of 3 μm were cut and hematoxylin and eosin was used for staining. Sections were examined using alight microscope(Olympus BX-50 Olympus Corporation, Tokyo, Japan).

### Biochemical analysis

#### Measurement of lipid peroxidation

Determination of malondialdehyde (MDA) level, as a measure of lipid peroxidation, in the granulation tissue was carried out according to the kit's instructions (Biodiagnostic, Egypt). The method is based upon the reaction between thiobarbituric acid and MDA in acidic medium at temperature of 95°C for 30 min to form thiobarbituric acid reactive product; the absorbance of the resultant pink product was measured at 534 nm [28, 29].

#### Estimation of reduced glutathione

Reduced glutathione was estimated according to the kit's instructions (Biodiagnostic, Egypt). The method is based upon the reduction of 5,5`dithiobis (2—nitrobenzoicacid) with GSH to produce a yellow compound. The reduced chromogen is directly proportional to GSH concentration and its absorbance can be measured at 405 nm [30].

### Statistical analysis

All data were expressed as mean ± SEM and analyzed by one-way ANOVA followed by Tukey’s test as a post hoc test. All statistical analyses were performed using the GraphPad Prism software (version 5.01, Inc., 2007, San Diego California USA). Probability values of less than 0.05 were considered statistically significant.

### Evaluation of drug interaction by CDI

The coefficient of drug interaction (CDI) was used to analyze the effect of drug combination on the wound contraction percent, MDA and GSH levels. For reduced (i.e. diminished function) effect, the used equation was CDI = AB/(A × B); and for increased effect (i.e. enhanced function), the used equation was CDI = (A × B)/AB, where AB is the ratio of the combination group to its control group; A or B is the ratio of the single essential oil to its control group. In the current study, combination index scale was defined as follows: CDI < 0.9: synergistic, CDI = 0.9–1.1: additive, CDI > 1.1 antagonistic.

## Results

### GC/MS analysis of essential oils

#### GC/MS analysis of the essential oil of *Melaleuca alternifolia*

**Fig 1:**
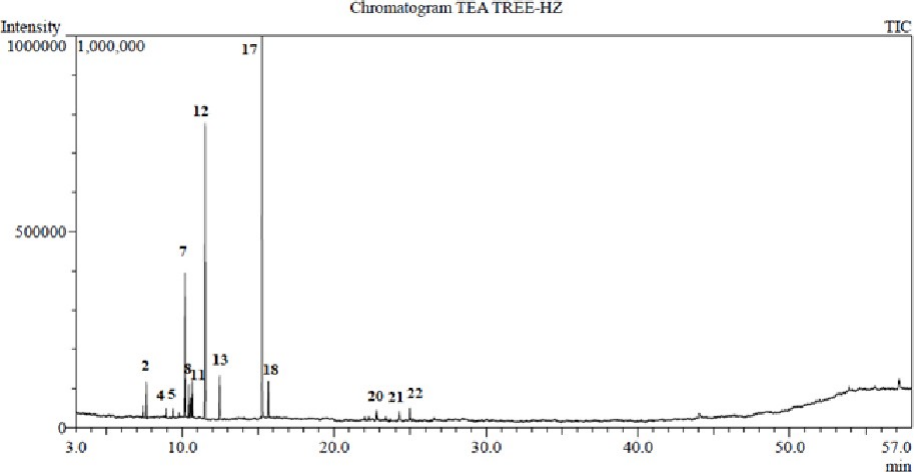
GC/MS chromatogram of the essential oil of *Melaleuca alternifolia* (Tea tree).

Tea tree oil exhibited a pale yellow color with characteristic spicy odor. Meanwhile, rosemary essential oil exhibited a light yellow color with a distinctive aromatic odor. Sixteen components were identified for *M*. *alternifolia* essential oil representing 100% of the total oil composition (Table 1, Fig 1). Oxygenated monoterpenes constituted almost half of the oil composition while the monoterpene hydrocarbons constituted 46.66%. The major components were terpinen-4-ol (45.23%), *γ*-terpinene (23.07%), *α*-terpinene (10.84%), terpinolene (3.50%), *α*-terpineol (2.95%), 1,8-cineole (2.88%) and *α*-pinene (2.55%).

**Table 1:** Chemical composition of the essential oils of *M*. *alternifolia* and *R*. *officinalis*.

Peak #	R_t_	Component	Molecular formula	*RI*_*exp*_^*a*^	*RI*_*lit*_^*b*^	Content (%)		Identification^e^
						MA^c^	RO^d^	
1	7.43	*α*-Thujene	C_10_H_16_	918	918	0.77	**-**	MS, RI
2	7.63	*α*-Pinene	C_10_H_16_	925	925	2.55	**13.94**	MS, RI
3	8.07	Camphene	C_10_H_16_	941	940	**-**	3.78	MS, RI
4	8.93	*β*-pinene	C_10_H_16_	972	972	0.71	1.28	MS, RI
5	9.39	*β*-Myrcene	C_10_H_16_	989	989	0.63	1.12	MS, RI
6	9.79	*α*-Phellandrene	C_10_H_16_	1003	1003	0.31	-	MS, RI
7	10.17	*α*-Terpinene	C_10_H_16_	1015	1015	**10.84**	-	MS, RI
8	10.43	*p*-Cymene	C_10_H_14_	1023	1023	2.51	2.68	MS, RI
9	10.55	*D*-Limonene	C_10_H_16_	1027	1027	**-**	3.01	MS, RI
10	10.56	Sylvestrene	C_10_H_16_	1028	1028	1.77	**-**	MS, RI
11	10.64	1,8-Cineole	C_10_H_18_O	1030	1030	2.88	**53.67**	MS, RI
12	11.51	*γ*-Terpinene	C_10_H_16_	1058	1058	**23.07**	**-**	MS, RI
13	12.44	Terpinolene	C_10_H_16_	1088	1088	3.50	**-**	MS, RI
14	12.81	Linalool	C_10_H_18_O	1100	1100	-	0.86	MS, RI
15	14.24	Camphor	C_10_H_16_O	1146	1146	**-**	**10.43**	MS, RI
16	14.92	Borneol	C_10_H_18_O	1167	1167	**-**	2.58	MS, RI
17	15.26	Terpinen-4-ol	C_10_H_18_O	1179	1179	**45.23**	-	MS, RI
18	15.66	*α*-Terpineol	C_10_H_18_O	1192	1192	2.95	2.07	MS, RI
19	22.27	Caryophyllene	C_15_H_24_	1424	1504	-	2.74	MS, RI
20	22.80	Alloaromadendrene	C_15_H_24_	1445	1445	0.86	-	MS, RI
21	24.27	Ledene (Viridiflorene)	C_15_H_24_	1503	1502	0.51	-	MS, RI
22	24.96	*δ*-Cadinene	C_15_H_24_	1530	1530	0.91	-	MS, RI
**Monoterpene hydrocarbons**						**46.66**	**25.81**	
**Oxygenated monoterpenes**						**51.06**	**69.61**	
**Sesquiterpene hydrocarbons**						**2.28**	**2.74**	
**Total identified components**						**100**	**98.16**	

^a^*RI*_*exp*_, Retention index determined experimentally on Rtx-5MS column

^b^*RI*_*lit*_, published retention indices

^*c*^*MA*, *Melaleuca alternifolia* essential oil

^*d*^*RO*,*Rosmarinus officinalis* essential oil

^e^Identification, was based on comparison of the compounds’ mass spectral data (MS) and retention indices (RI) with those of NIST Mass Spectral Library (2011), Wiley Registry of Mass Spectral Data 8^th^edition and literature.

#### GC/MS analysis of the essential oil of *Rosmarinus officinalis*

**Fig 2:**
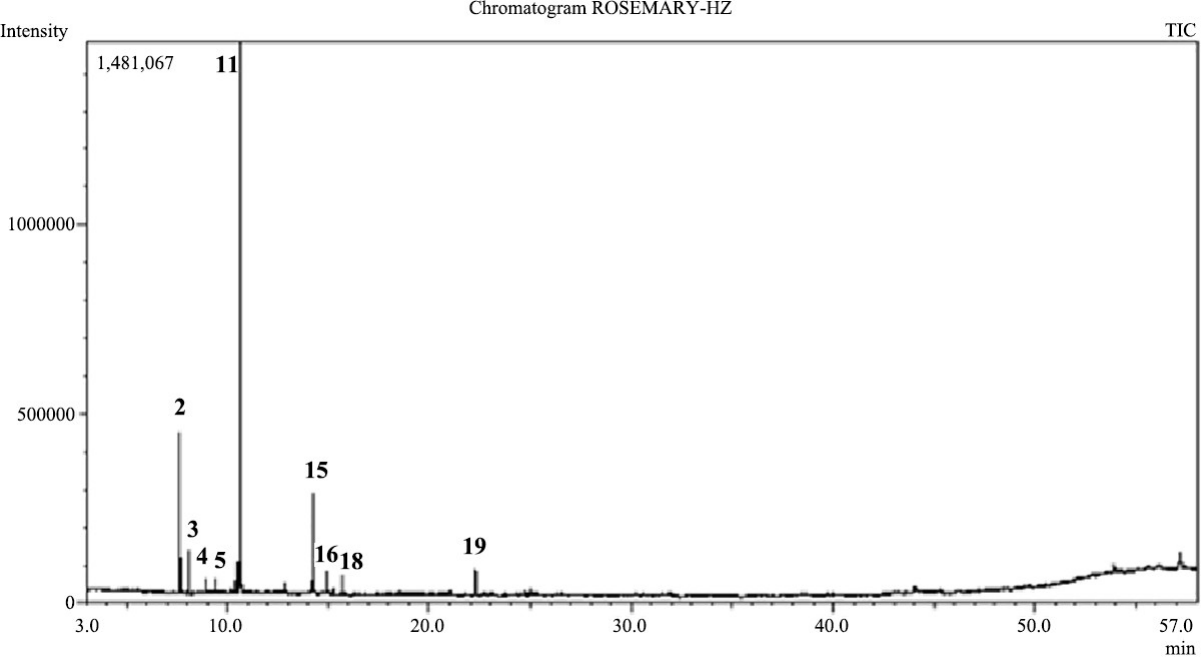
GC/MS chromatogram of the essential oil of *Rosmarinus officinalis*.

Twelve components were identified for *R*. *officinalis* essential oil representing 98.16% of the total oil (Table 1, Fig 2). The major components were1,8-cineole (53.67%), *α*-pinene (13.94%), camphor (10.43%) and camphene (3.78%). Oxygenated monoterpenes constituted 69.61% on the other hand monoterpene hydrocarbons constituted 25.81%.

### Effect of topical application of different treatments on wound contraction

**Fig 3:**
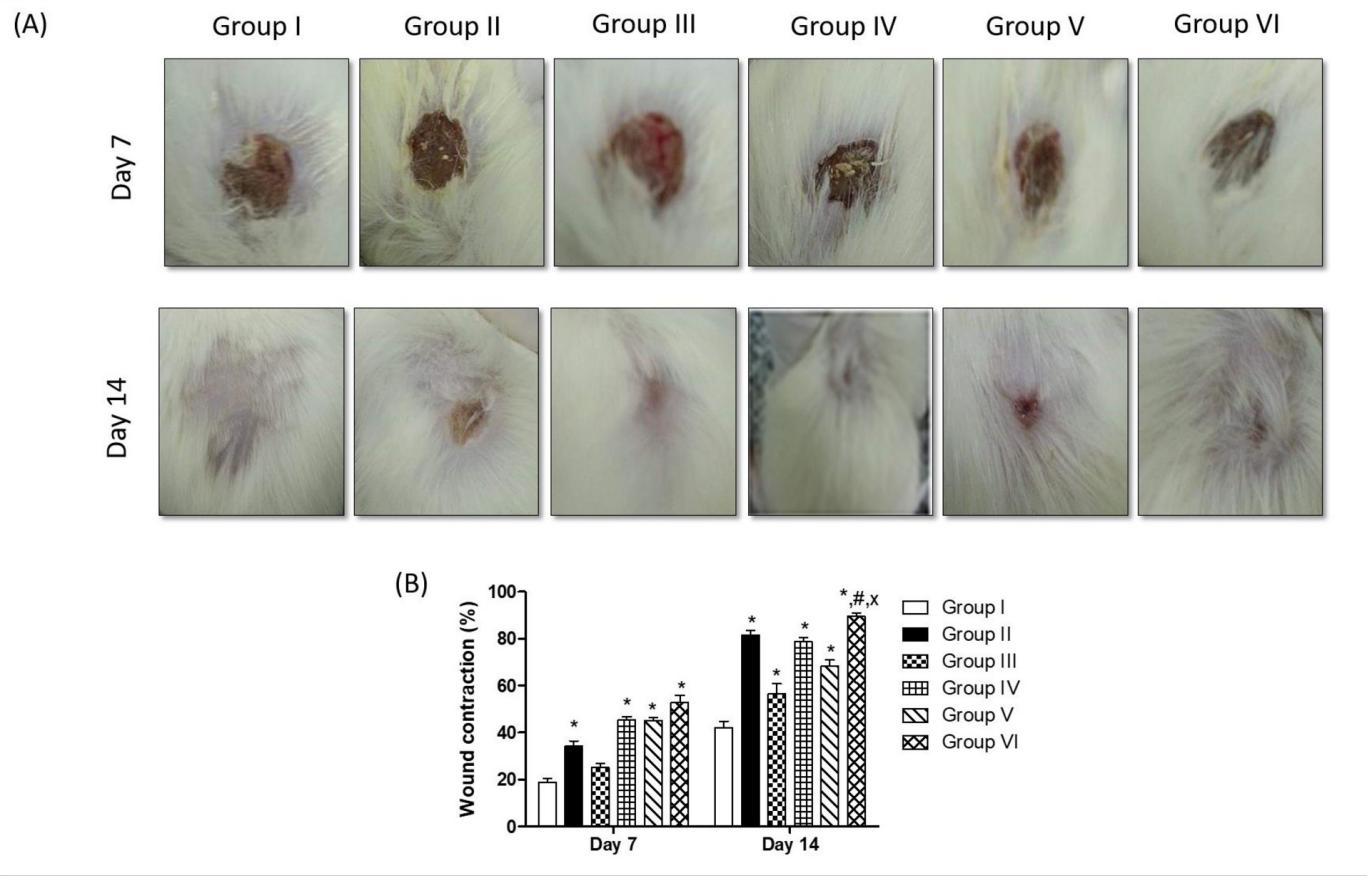
(A) Representative photographs showing wound closure in rats with topical application of different treatments on day 7 and day 14. (B) Effects of different treatments on percent wound closure on different days in rats (wound contraction, %). Values are given as mean ± SEM (n = 6). *, #, x indicates p<0.05, compared to Group I, IV and V, respectively. Results were analyzed using one-way ANOVA followed by Tukey’s test.

Wound contraction was calculated as percent reduction in wound area [31] on the 7^th^ and 14^th^ days of the experiment. As illustrated in Fig 3, topical application of tea tree, rosemary and tea tree and rosemary mixture resulted in a significant increase in the wound contraction percent by 2.43, 2.41 and 2.82 fold, respectively, compared to the negative control group, on day 7 of the experiment. Moreover, the wound contraction percent was significantly higher in these groups compared to the positive control group. Furthermore, topical application of tea tree, rosemary and tea tree and rosemary mixture resulted in a significant increase in the wound contraction percent by 1.87, 1.63 and 2.13 fold, respectively, compared to the negative control group, on day 14 of the experiment. Noteworthy, topical application of the tea tree and rosemary mixture resulted in a significant increase in the wound contraction percent compared to either groups separately treated with individual essential oil and the result of the mixture was comparable to that of the positive control group.

### Histopathology

#### Microscopic examination of different skin wound samples on day 7

**Fig 4:**
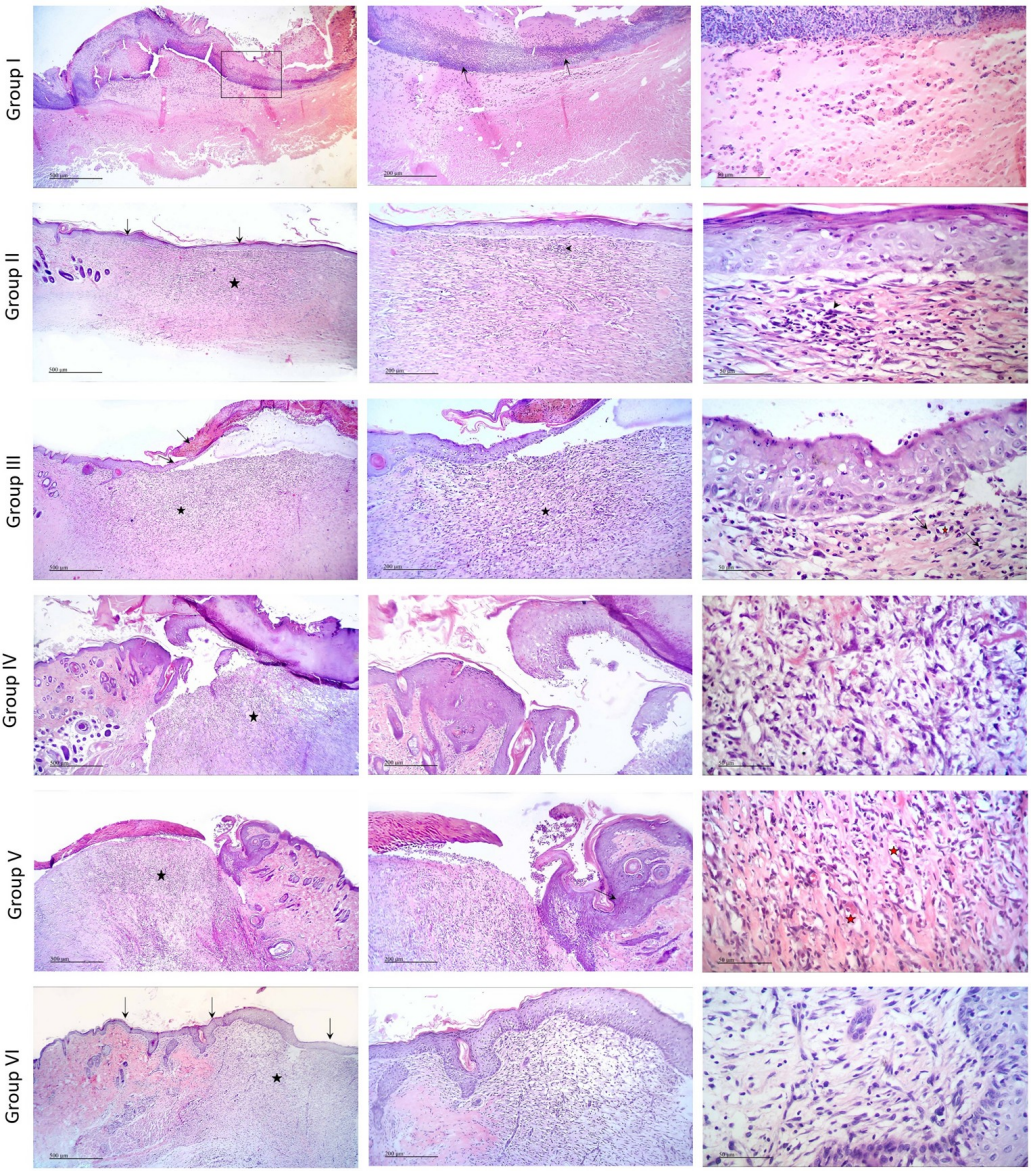
Histopathological view of wound healing and epidermal/dermal re-modeling in the groups administered different treatments on day 7 at magnification 4x, 10x and 40x.

As illustrated in Fig 4 and scored in Table 2, microscopic examination revealed that the negative control samples (Group I) showed retarded wound healing with severe necrosis of the dermal and epidermal region, diffuse inflammatory cells infiltrations obscured the dermo-epidermal junction(arrow). The positive control (Group II) demonstrated complete re-epithelialization (arrow) with underlying large area of dermal layer occupied by highly cellular granulation tissue (star) with focal inflammatory cell infiltrations at dermo-epidermal junction (arrow heads), newly formed blood vessels with few activated hair follicles. Plain samples (Group III) showed incomplete re-epithelialization with ulceration and scab formation (arrow) with many tissue debris infiltrated with inflammatory cells. Wide area of granulation tissue formation was observed in the dermis (star), rich in blood vessels (red star), fibroblasts and inflammatory cells infiltrations (arrow head).

**Table 2:** Wound healing processes and healing phases of the animals administered different treatments on day 7.

Groups	Wound healing processes						Healing phases		
	S	U	RE	FP	CD	IC	I	P	R
**I**	+++	+++	-	++	++	+++	+++	+++	-
**II**	++	+	+	+/++	++	++	++	++/+++	++
**III**	+++	++/+++	-/+	++	++	++/+++	++	+++	-
**IV**	++	+	-/+	+	+	++	+	++	+
**V**	++	++	-/+	++	++	++	+/++	++	+
**VI**	++	+	+	+	++	++	++	+++	++

HE (Hematoxylin &Eosin) stained sections were scored as mild (+), moderate (++) and severe (+++) for epidermal and/or dermal re-modeling. S: Scab, U: Ulcus, RE: Re-epithelization, FP:Fibroblast proliferation, CD: Collagen depositions, IC: Inflammatory cells infiltration, I: Inflammation phase, P: Proliferation phase, R: Re-modeling phase.

Tea tree oil-treated group (Group IV) and rosemary oil-treated group (Group V) demonstrated retarded wound healing process showing incomplete re-epithelialization with ulcer formation and adjacent acanthosis (arrow). Scab formation was observed over highly cellular dermal granulation tissue (star) with diffuse inflammatory cells inflations and rich in blood capillaries (red stars). Interestingly, tea tree and rosemary oils based chitosan topical preparation-treated group (Group VI) was closely related to the positive control group. Samples demonstrated complete re-epithelialization (arrow) with underlying large area of dermal layer occupied by highly cellular granulation tissue (star) together with many inflammatory cells infiltrations and newly formed blood vessels with few activated hair follicles.

#### Microscopic examination of different skin wound samples on day 14

**Fig 5:**
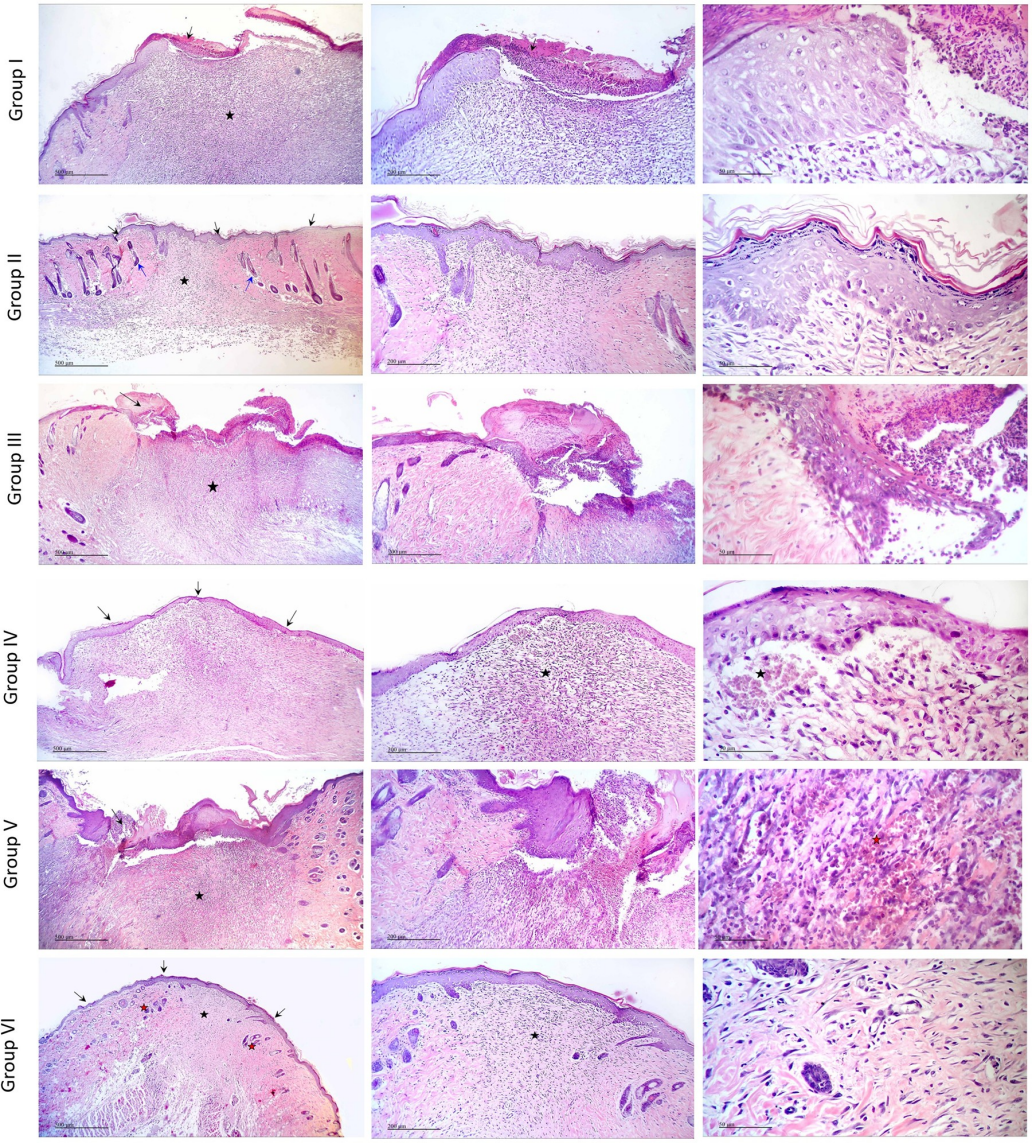
Histopathological view of wound healing and epidermal/dermal re-modeling in the groups administered different treatments on day 14 at magnification 4x, 10x and 40x.

As illustrated in Fig 5 and scored in Table 3, on day 14, microscopic examination of the negative control (Group I) and plain groups samples (Group III) showed retarded wound healing, ulceration and scab formation with tissue debris and inflammatory cells infiltrates (arrow). Wide diffuse highly cellular granulation tissue (star)was observed in the dermal layer rich in inflammatory cells and many activated fibroblasts with newly formed blood vessels. Positive control samples (Group II) demonstrated accelerated wound healing process with complete re-epithelialization (black arrows) and mild vacuolar changes in basal keratenocytes of epidermis. Higher amount of mature collagen fibers formation accompanied with activated hair follicles (blue arrows) and lesser area of more cellular less fibrous granulation tissue (star) with many fibroblasts were observed.

**Table 3:** Wound healing processes and healing phases of the animals administered different treatments on day 14.

Groups	Wound healing processes						Healing phases		
	S	U	RE	FP	CD	IC	I	P	R
**I**	++	++	-/+	++	++	++	++	+++	-/+
**II**	+	-	+++	+/++	++	+	+	++	+++
**III**	++	++/+++	+	++	++	++	++	++/+++	+
**IV**	++	+	++	++	+	+/++	+	+/++	++
**V**	++	++	-/+	++	++	+/++	+/++	++	+/++
**VI**	+	-/+	+++	++	++	-/+	-/+	++	+++

HE (Hematoxylin &Eosin) stained sections were scored as mild (+), moderate (++) and severe (+++) for epidermal and/or dermal re-modeling. S: Scab, U: Ulcus, RE: Re-epithelization, FP:Fibroblast proliferation, CD: Collagen depositions, IC: Inflammatory cells infiltration, I: Inflammation phase, P: Proliferation phase, R: Re-modeling phase.

Tea tree-treated samples (Group IV) showed complete re-epithelialization of epidermal layer (arrow). Wide area of highly cellular less fibrous granulation tissue in the dermis was detected, rich in small blood capillaries. Focal areas of sub-epithelial hemorrhages were observed (star). Rosemary-treated samples (Group V) showed incomplete re-epithelialization of epidermal layer. Ulceration, scab formation, with tissue depress and inflammatory cells infiltrates (arrow). Smaller diffuse area showing highly cellular granulation tissue (star) in dermal layer, rich in inflammatory cells with focal areas of hemorrhages (red star).Higher numbers of active hair follicles were observed in wounded area sides. Tea tree and rosemary oils based chitosan topical preparation-treated group (Group VI) showed more advanced wound healing process resembling the positive control samples. Complete re-epithelialization of the epidermal layer (arrows)was perceived with few degenerative changes in some keratenocytes. Dermal layer showed smaller area of granulation tissue (black star) rich in fibroblasts and inflammatory cells with higher area of mature collagen fibers formation accompanied with many activated hair follicles and minimal inflammatory cells infiltrates on both sides of the wound area (red star).

### Estimation of thiobarbituric acid reactive substances (TBARS) level expressed as malondialdehyde (MDA)

**Fig 6:**
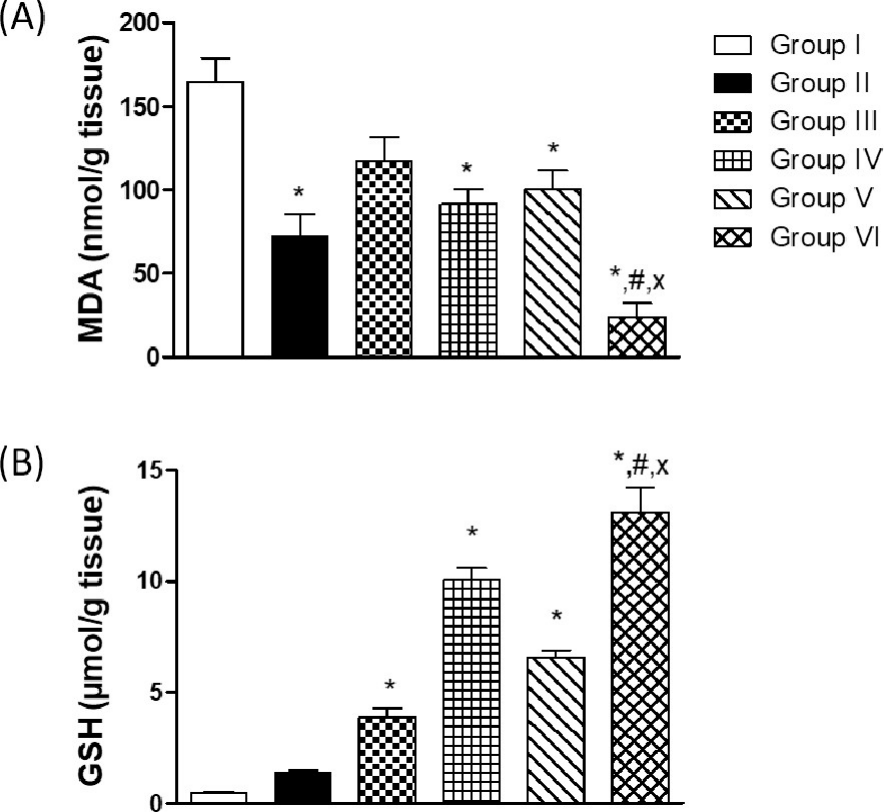
Thiobarbituric acid reactive substances (TBARS) level expressed as malondialdehyde (MDA) (A) and reduced glutathione in the wound tissues (B). Values are given as mean ± SEM (n = 6). *,#,x indicates p<0.05, compared to Group I, IV and V, respectively. Results were analyzed using one-way ANOVA followed by Tukey’s test.

MDA level was significantly reduced in the wound granulation tissue isolated from the positive control group by 56.10% compared to the negative control group. Topical application of tea tree and rosemary resulted in a significant reduction in MDA level by 44.44 and 38.97%, respectively, compared to the negative control group. Noteworthy, the MDA level in the tea tree and rosemary mixture-treated group was comparable to that of the positive control group (Fig 6).

### Estimation of reduced glutathione in the wound tissues

The level of GSH was significantly elevated in the wound granulation tissue isolated from the plain group by 8.05 fold compared to the negative control group. No significant change was observed in the GSH level in the positive control group compared to the negative control group. The level of GSH in the groups treated by tea tree and rosemary was significantly elevated by 20.74 and 13.60 fold, respectively, compared to the negative control group. Noteworthy, the GSH level in the tea tree and rosemary mixture-treated group was significantly elevated by 27.05 fold compared to the negative control group (Fig 6).

### Evaluation of CDI for the combination

To investigate the type of interaction for the combination, CDI was calculated for the percent of wound contraction as well as the effect on MDA and GSH levels. Results are listed in Table 4 demonstrating synergistic and antagonistic effects.

**Table 4:** Nature of interaction between tea tree and rosemary essential oils as determined by CDI.

Parameter	CDI	Effect of combination
**Wound contraction percent on day 7**	2.076	Antagonistic
**Wound contraction percent on day 14**	1.42	Antagonistic
**MDA level**	0.44	Synergistic
**GSH level**	10.43	Antagonistic

## Discussion

The wound healing potential of three chitosan-based formulations loaded with either tea tree essential oil or rosemary essential oil or a mixture of both oils was investigated in the present study. In order to reveal the combined interaction of the ingredients in the formulation, each material was investigated individually for its wound healing potential. Stages in wound healing processes (inflammation, proliferation, and remodeling) were observed and recorded within the different groups on days 7 and 14 of the experiment. Wound healing was evaluated using the antioxidant markers *viz*. MDA and reduced GSH levels.

Chitosan is well-known for its favorable wound healing capacity by enhancing rapid dermal regeneration, in addition to bacteriostatic, and accelerated wound healing properties [32]. Moreover, chitosan enhanced re-epithalization and normal skin regeneration in open wounds [33]. Nevertheless, several studies reported chitosan films prepared with chitosan concentration (1–3%) [24, 34, 35] to have acceptable mechanical properties, moisture content and particle size. In line with the previously reported favorable effects of chitosan, the present study showed enhanced wound contraction in the plain group (chitosan only-treated group), in addition to increased levels of reduced glutathione in the granulation tissue isolated from this group.

*M*. *alternifolia* essential oil displayed wide applications in dermatology, effectively treating multiple skin diseases and infections [36]. Itis currently being incorporated in a plethora of skin care products owing to its antimicrobial properties, primarily due toterpinen-4-ol, its major constituent [37]. Several reports showed that the antioxidant, anti-inflammatory, and antimicrobial properties of tea tree oil contribute to its wound healing potential [38]. Furthermore, tea tree oil inhibited lipopolysaccharide-induced production of tumor necrosis factor-alpha, interleukin-1*β* and IL-10. It has been shown that terpinen-4-ol, but not *α*-terpineol or 1,8-cineole,was responsible for reducing the production of inflammatory mediators [39].

As stated by the International Standard Organization (ISO 4730), *M*.*alternifolia* commercial essential oil should have a minimal of 30.0% composition of terpinen-4-ol, meanwhile, the level of 1,8-cineole should not exceed 15.0% in order to maintain antiseptic property [40]. Chemical composition of *M*. *alternifolia* essential oil used in this study, complied with ISO 4730, whereterpinen-4-ol represented 45.23% of the total oil composition, whereas 1,8-cineole represented only 2.88%.

Rosemary essential oil is reported to possess antibacterial, antifungal, antioxidant and anti-inflammatory properties [41] as well as wound healing potential [12]. *R*. *officinalis* essential oil was shown to possess significant wound healing effect, when applied topically to the wound of diabetic mice, affecting multiple stages of the healing process [12]. The present study, in harmony with previous studies, showed improved wound contraction and enhanced oxidative stress status (evidenced by reduced lipid peroxidation and increased GSH levels) in the tea tree and rosemary-treated groups. Moreover, histopathological examination showed enhanced re-epithelialization in these groups as compared to the negative control group. Interestingly, topical application of a mixture of tea tree and rosemary oils demonstrated complete re-epithelialization with activated hair follicles, an effect which was comparable to that of the positive control group. The high percentage of oxygenated monoterpenes in both tea tree (51.06%), and rosemary (69.61%) essential oils, play an important role in the antioxidant, and wound healing potential observed herein.

While CDI determination could be a valuable tool providing information on the nature of interactions between drug combinations, it should be cautiously considered, especially in the absence of dose dependent studies for the combination, where the present study investigated the wound healing effect of tea tree and rosemary essential oils at a single concentration (10%). Moreover, the effect could be capacity limited, where simply a synergistic effect could not be achieved, resulting in an antagonistic effect. For example, the CDI for the effect of the mixture of tea tree and rosemary essential oils on the percent of wound contraction and GSH level was calculated to be antagonistic, although the effect was significantly higher in the group treated with the essential oil mixture as compared to the groups treated with either essential oil alone. In the absence of dose/concentration dependent studies, it can be speculated that the observed effect in the mixture could have reached maximum capacity to increase the percent wound contraction or GSH level in the granulation tissue of wounded animals.

## Conclusion

From this study, it can be concluded that the incorporation of tea tree and rosemary essential oils in chitosan-based preparations in appropriate combination could efficiently promote different stages of wound healing. In addition, it decreased oxidative stress in the wound area.

## Supporting information

S1 Fig EI/MS spectrum of compound (1) identified as *α*-Thujene in the essential oil of *M*. *alternifolia*. (PDF)

S2 Fig EI/MS spectrum of compound (2) identified as *α*-Pinene in the essential oils of *M*. *alternifolia* and *R*. *officinalis*. (PDF)

S3 Fig EI/MS spectrum of compound (3) identified as Camphene in the essential oil of *R*. *officinalis*. (PDF)

S4 Fig EI/MS spectrum of compound (4) identified as *β*-pinene in the essential oils of *M*. *alternifolia* and *R*. *officinalis*. (PDF)

S5 Fig EI/MS spectrum of compound (5) identified as *β-*Myrcene in the essential oils of *M*. *alternifolia* and *R*. *officinalis*. (PDF)

S6 Fig EI/MS spectrum of compound (6) identified as *α*-Phellandrene in the essential oil of *M*. *alternifolia*. (PDF)

S7 Fig EI/MS spectrum of compound (7) identified as *α-*Terpinene in the essential oil of *M*. *alternifolia*. (PDF)

S8 Fig EI/MS spectrum of compound (8) identified as *p*-Cymene in the essential oils of *M*. *alternifolia* and *R*. *officinalis*. (PDF)

S9 Fig EI/MS spectrum of compound (9) identified as *D*-Limonene in the essential oil of *R*. *officinalis*. (PDF)

S10 Fig EI/MS spectrum of compound (10) identified as Sylvestrene in the essential oil of *M*. *alternifolia*. (PDF)

S11 Fig EI/MS spectrum of compound (11) identified as 1,8-Cineolein the essential oils of *M*. *alternifolia* and *R*. *officinalis*. (PDF)

S12 Fig EI/MS spectrum of compound (12) identified as *γ*-Terpinene in the essential oil of *M*. *alternifolia*. (PDF)

S13 Fig EI/MS spectrum of compound (13) identified as Terpinolene in the essential oil of *M*. *alternifolia*. (PDF)

S14 Fig EI/MS spectrum of compound (14) identified as Linalool in the essential oil of *R*. *officinalis*. (PDF)

S15 Fig EI/MS spectrum of compound (15) identified as Camphor in the essential oil of *R*. *officinalis*. (PDF)

S16 Fig EI/MS spectrum of compound (16) identified as Borneol in the essential oil of *R*. *officinalis*. (PDF)

S17 Fig EI/MS spectrum of compound (17) identified as Terpinen-4-ol in the essential oil of *M*. *alternifolia*. (PDF)

S18 Fig EI/MS spectrum of compound (18) identified as *α*-Terpineol in the essential oils of *M*. *alternifolia* and *R*. *officinalis*. (PDF)

S19 Fig EI/MS spectrum of compound (19) identified as Caryophyllene in the essential oil of *R*. *officinalis*. (PDF)

S20 Fig EI/MS spectrum of compound (20) identified as Alloaromadendrene in the essential oil of *M*. *alternifolia*. (PDF)

S21 Fig EI/MS spectrum of compound (21) identified as Ledene in the essential oil of *M*. *alternifolia*. (PDF)

S22 Fig EI/MS spectrum of compound (22) identified as *δ*-Cadinene in the essential oil of *M*. *alternifolia*. (PDF)

S23 Fig Graphical abstract. (TIF)

## Abbreviations

GC/MS: Gas chromatography/Mass spectrometry
M. alternifolia: *Melaleuca alternifolia*
R. officinalis: *Rosmarinus officinalis*
CS: Chitosan
PVA: Polyvinyl alcohol
RI: retention index
DI H_2_O: deionized water
MDA: malondialdehyde
TBARS: thiobarbituric acid reactive substance
GSH: reduced glutathione
CDI: Coefficient of Drug Interaction

## References

[1] Budovsky A, Yarmolinsky L, Ben-Shabat S.Effect of medicinal plants on wound healing. Wound. Repair Regen. 2015; 23(2): 171–183. 10.1111/wrr.12274 25703533

[2] Kasuya A, Tokura Y.Attempts to accelerate wound healing. J Dermatol. Sci. 2014; 76(3): 169–172. 10.1016/j.jdermsci.2014.11.001 25468357

[3] Woollard AC, Tatham KC, Barker S.The influence of essential oils on the process of wound healing: a review of the current evidence. J Wound. Care. 2007; 16(6): 255–257. 10.12968/jowc.2007.16.6.27064 17722522

[4] Carson CF, Hammer KA, Riley TV. *Melaleuca alternifolia* (Tea Tree) oil: a review of antimicrobial and other medicinal properties. Clin Microbiol Rev. 2006; 19(1): 50–62. 10.1128/CMR.19.1.50-62.2006 16418522 PMC1360273

[5] Drury S. Tea Tree Oil: A Medicine Kit in a Bottle. Random House 2011.

[6] Hammer K. Treatment of acne with tea tree oil *(Melaleuca)* products: a review of efficacy, tolerability and potential modes of action. International journal of antimicrobial agents. 2015; 45(2): 106–110. 10.1016/j.ijantimicag.2014.10.011 25465857

[7] Syed TA, Qureshi ZA, Ali SM, Ahmad S, Ahmad SA. Treatment of toenail onychomycosis with 2% butenafine and 5% *Melaleuca alternifolia* (tea tree) oil in cream. Tropical Medicine & International Health.1999; 4(4): 284–287. 10357864 10.1046/j.1365-3156.1999.00396.x

[8] Satchell AC, Saurajen A, Bell C, Barnetson R. Treatment of interdigital *tinea pedis* with 25% and 50% tea tree oil solution: A randomized, placebo-controlled, blinded study. Australian Journal of Dermatology. 2002; 43(3): 175–178. 10.1046/j.1440-0960.2002.00590.x 12121393

[9] Yadav E, Kumar S, Mahant S, Khatkar S, Rao R. Tea tree oil: a promising essential oil. Jessent. oil research. 2017; 29(3): 201–213.

[10] Rašković A, Milanović I, Pavlović N, Ćebović T, Vukmirović S, Mikov M. Antioxidant activity of rosemary (*Rosmarinus officinalis* L.) essential oil and its hepatoprotective potential. BMC Complementary and Alternative Medicine. 2014; 14(1): 225. 25002023 10.1186/1472-6882-14-225 PMC4227022

[11] Yu MH, Choi JH, Chae IG, Im HG, Yang SA, More K, Lee IS, Lee J. Suppression of LPS-induced inflammatory activities by *Rosmarinus officinalis* L. Food Chemistry. 2013; 136(2): 1047–1054. 10.1016/j.foodchem.2012.08.085 23122161

[12] Abu-Al-Basal MA.Healing potential of *Rosmarinus officinalis* L. on full-thickness excision cutaneous wounds in alloxan-induced-diabetic BALB/c mice. J Ethnopharmacol. 2010;131(2): 443–50. 10.1016/j.jep.2010.07.007 20633625

[13] de Medeiros Barbosa I, da Costa Medeiros JA, de Oliveira KÁR, Gomes-Neto NJ, Tavares JF, Magnani M, de Souza EL. Efficacy of the combined application of oregano and rosemary essential oils for the control of *Escherichia coli*, *Listeria monocytogenes* and *Salmonella Enteritidis* in leafy vegetables. Food control. 2016; 59: 468–477.

[14] Fiume MM, Bergfeld WF, Belsito DV, Hill RA, Klaassen CD, Liebler DC, et al Safety Assessment of *Rosmarinus officinalis* (Rosemary)-Derived Ingredients as Used in Cosmetics. International Journal of Toxicology. 2018 37(3-suppl): 12S–50S. 10.1177/1091581814545247 25297908

[15] Saporito F, Sandri G, Bonferoni MC, Rossi S, Boselli C, Icaro CA, et al Essential oil-loaded lipid nanoparticles for wound healing. Int. J Nanomedicine. 2018; 13: 175–186. 10.2147/IJN.S152529 29343956 PMC5747963

[16] Patrulea V, Ostafe V, Borchard G, Jordan O.Chitosan as a starting material for wound healing applications. Eur. J Pharm. Biopharm. 2015; 97(Pt B): 417–426. 10.1016/j.ejpb.2015.08.004 26614560

[17] Pelissari FM, Grossmann MV, Yamashita F, Pineda EA. Antimicrobial, mechanical, and barrier properties of cassava starch-chitosan films incorporated with oregano essential oil. J Agric Food Chem. 2009; 57(16): 7499–504. 10.1021/jf9002363 19627142

[18] Raafat D, Sahl HG.Chitosan and its antimicrobial potential-a critical literature survey. Microb. Biotechnol. 2009; 2(2): 186–201. 10.1111/j.1751-7915.2008.00080.x 21261913 PMC3815839

[19] Delaquis PJ, Stanich K, Girard B, Mazza G. Antimicrobial activity of individual and mixed fractions of dill, cilantro, coriander and eucalyptus essential oils. Int. J Food Microbiol. 2002; 74(1–2): 101–109. 11929164 10.1016/s0168-1605(01)00734-6

[20] Amin M, abdel-Raheem I. Accelerated wound healing and anti-inflammatory effects of physically cross linked polyvinyl alcohol-chitosan hydrogel containing honey bee venom in diabetic rats. Arch. Pharm. Res. 2014; 37(8): 1016–1031. 10.1007/s12272-013-0308-y 24293065

[21] Adams RP. Identification of essential oil components by gas chromatography/mass spectorscopy: Allured Publishing Corporation 2007.

[22] Elkady WM, Ayoub IM.Chemical profiling and antiproliferative effect of essential oils of two *Araucaria* species cultivated in Egypt. Industrial Crops and Products. 2018;118:188–195.

[23] Labib RM, Youssef FS, Ashour ML, Abdel Daim MM, Ross SA. Chemical composition of *Pinus roxburghii* bark volatile oil and validation of its anti-Inflammatory activity using molecular modelling and bleomycin-induced inflammation in *Albino* mice. Molecules. 2017; 22(9): 1384. 10.3390/molecules22091384 PMC6151475 28850077

[24] Wang L, Liu F, Jiang Y, Chai Z, Li P, Cheng Y, et al Synergistic antimicrobial activities of natural essential oils with chitosan films. J Agric. Food Chem. 2011; 59(23): 12411–12419. 10.1021/jf203165k 22034912

[25] Ponrasu T, Suguna L.Efficacy of *Annona squamosa* on wound healing in streptozotocin-induced diabetic rats. Int Wound J. 2012; 9(6): 613–23. 10.1111/j.1742-481X.2011.00924.x 22233431 PMC7950626

[26] Kant V, Gopal A, Pathak NN, Kumar P, Tandan SK, Kumar D. Antioxidant and anti-inflammatory potential of curcumin accelerated the cutaneous wound healing in streptozotocin-induced diabetic rats. International immunopharmacology. 2014; 20(2): 322–330. 10.1016/j.intimp.2014.03.009 24675438

[27] Sardari K, Kakhki EG, Mohri M.Evaluation of wound contraction and epithelializationafter subcutaneous administration of Theranekron in cows. Comp Clin Pathol. 2007; 16: 197–200.

[28] Ohkawa H, Ohishi N, Yagi K.Assay for lipid peroxides in animal tissues by thiobarbituric acid reaction. Analytical biochemistry. 1979; 95(2): 351–358. 10.1016/0003-2697(79)90738-3 36810

[29] Kei S. Serum lipid peroxide in cerebrovascular disorders determined by a new colorimetric method. Clinica chimica acta. 1978; 90(1): 37–43. 10.1016/0009-8981(78)90081-5 719890

[30] Beutler E, Duron O, Kelly M.Colorimetric method for determination of glutathione reductase concentration. Journal of Laboratory and Clinical Medicine. 1963; 61: 882 13967893

[31] Saha K, Mukherjee PK, Das J, Pal M, Saha BP. Wound healing activity of *Leucas lavandulaefolia* Rees. Journal of Ethnopharmacology. 1997; 56(2): 139–144. 9174975 10.1016/s0378-8741(97)01522-5

[32] Shi C, Zhu Y, Ran X, Wang M, Su Y, Cheng T. Therapeutic potential of chitosan and its derivatives in regenerative medicine. J Surg Res. 2006;133(2): 185–92. 10.1016/j.jss.2005.12.013 16458923

[33] Kweon DK, Song SB, Park YY. Preparation of water-soluble chitosan/heparin complex and its application as wound healing accelerator. Biomaterials. 2003; 24(9): 1595–601. 12559819 10.1016/s0142-9612(02)00566-5

[34] Tripathi S, Mehrotra G, Dutta P. Physicochemical and bioactivity of cross-linked chitosan–PVA film for food packaging applications. International Journal of Biological Macromolecules. 2009; 45(4): 372–376. 10.1016/j.ijbiomac.2009.07.006 19643129

[35] Caner C, Vergano P, Wiles J.Chitosan film mechanical and permeation properties as affected by acid, plasticizer, and storage. Journal of food science. 1998; 63(6): 1049–1053.

[36] Pazyar N, Yaghoobi R, Bagherani N, Kazerouni A. A review of applications of tea tree oil in dermatology. International Journal of Dermatology. 2013;52(7): 784–790. 10.1111/j.1365-4632.2012.05654.x 22998411

[37] Farag RS, Shalaby AS, El-Baroty GA, Ibrahim NA, Ali MA, Hassan EM. Chemical and biological evaluation of the essential oils of different *Melaleuca* species. Phytotherapy Research. 2004; 18(1): 30–35. 10.1002/ptr.1348 14750197

[38] Chin KB, Cordell B. The effect of tea tree oil (*Melaleuca alternifolia*) on wound healing using a dressing model. J Altern Complement Med. 2013; 19(12): 942–5. 10.1089/acm.2012.0787 23848210

[39] Brand C, Ferrante A, Prager RH, Riley TV, Carson CF, Finlay-Jones JJ. et al The water-soluble components of the essential oil of *Melaleuca alternifolia* (tea tree oil) suppress the production of superoxide by human monocytes, but not neutrophils, activated *in vitro*. Inflam Res. 2001; 50(4): 213–9. 10.1007/s000110050746 11392609

[40] Silva CJ, Barbosa LCA, Maltha CRA, Pinheiro AL, Ismail FMD. Comparative study of the essential oils of seven *Melaleuca* (Myrtaceae) species grown in Brazil. Flavour and Fragrance Journal. 2007; 22(6): 474–478.

[41] Takaki I, Bersani-Amado LE, Vendruscolo A, Sartoretto SM, Diniz SP, Bersani-Amado CA, et al Anti-inflammatory and antinociceptive effects of *Rosmarinus officinalis* L. essential oil in experimental animal models. J Med Food. 2008; 11(4): 741–6. 10.1089/jmf.2007.0524 19053868

