# Supplementary material for: Appraisal on the wound healing potential of Melaleuca alternifolia and Rosmarinus officinalis L. essential oil-loaded chitosan topical preparations
Source: PLoS One. 2019 Sep 16;14(9):e0219561. doi: 10.1371/journal.pone.0219561 (PMC6746351; doi:10.1371/journal.pone.0219561)
Supplement: S1 Fig — (PDF) [file pone.0219561.s001.pdf]

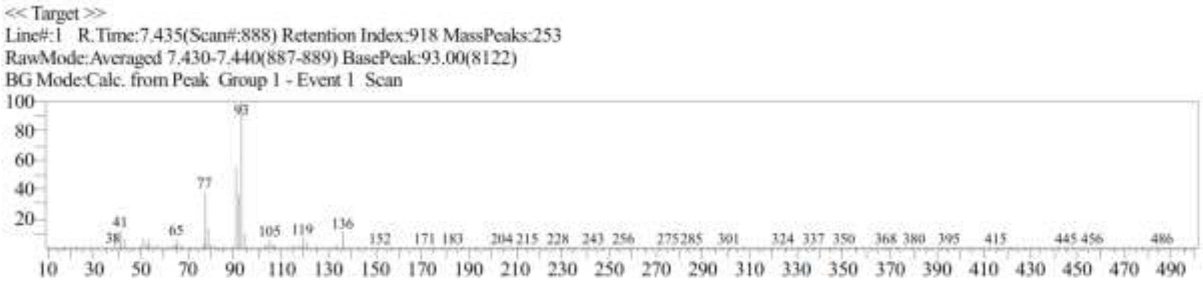

S1 Fig. EI/MS spectrum of compound (1) identified as α-Thujene in the essential oil of *M. alternifolia*
