# Supplementary figures and images for: Appraisal on the wound healing potential of Melaleuca alternifolia and Rosmarinus officinalis L. essential oil-loaded chitosan topical preparations
Source: PLoS One. 2019 Sep 16;14(9):e0219561. doi: 10.1371/journal.pone.0219561 (PMC6746351; doi:10.1371/journal.pone.0219561)

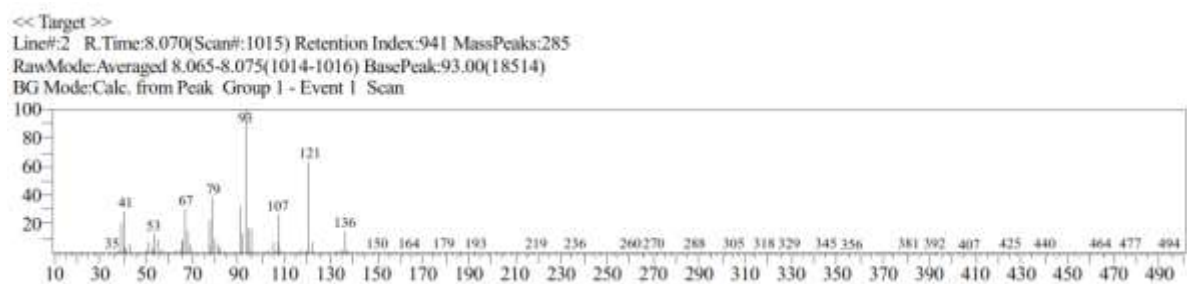

**S3 Fig. EI/MS spectrum of compound (3) identified as Camphene in the essential oil of *R. officinalis***

Supplement: S3 Fig — (PDF) [file pone.0219561.s003.pdf]

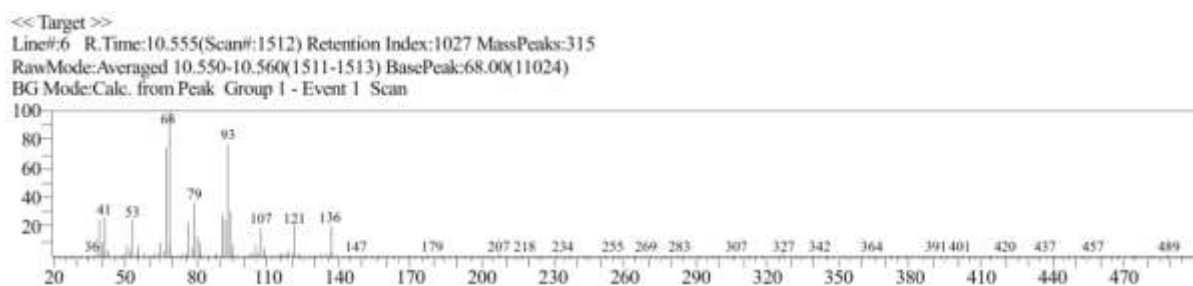

**S9 Fig. EI/MS spectrum of compound (9) identified as *D*-Limonene in the essential oil of *R. officinalis***

Supplement: S9 Fig — (PDF) [file pone.0219561.s009.pdf]

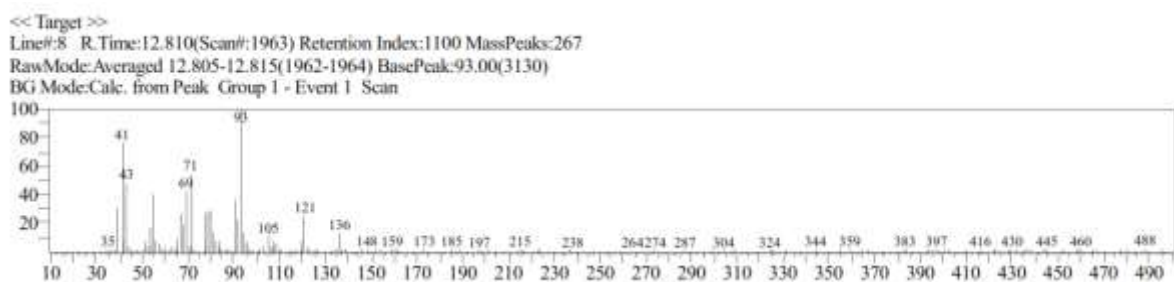

**S14 Fig. EI/MS spectrum of compound (14) identified as Linalool in the essential oil of *R. officinalis***

Supplement: S14 Fig — (PDF) [file pone.0219561.s014.pdf]

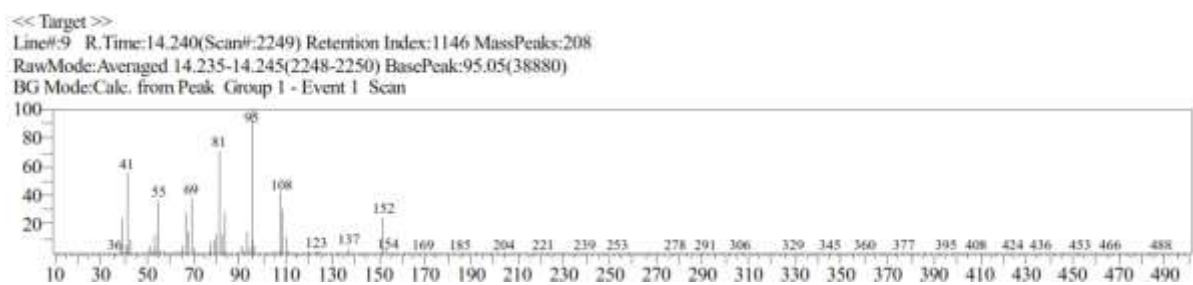

**S15 Fig. EI/MS spectrum of compound (15) identified as Camphor in the essential oil of *R. officinalis***

Supplement: S15 Fig — (PDF) [file pone.0219561.s015.pdf]

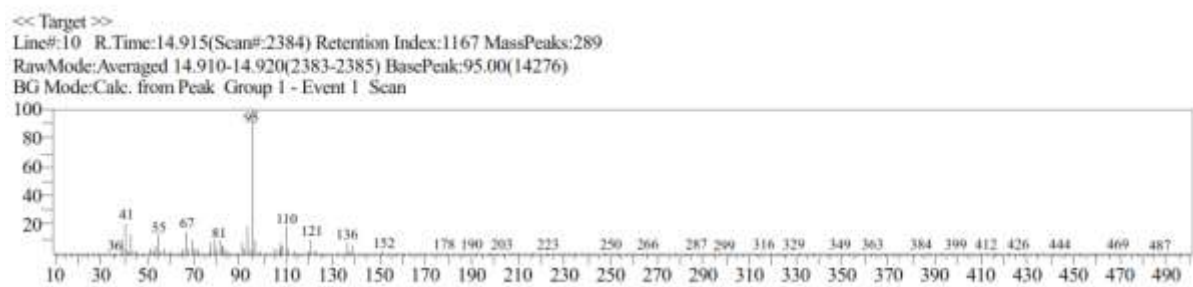

**S16 Fig. EI/MS spectrum of compound (16) identified as Borneol in the essential oil of *R. officinalis***

Supplement: S16 Fig — (PDF) [file pone.0219561.s016.pdf]

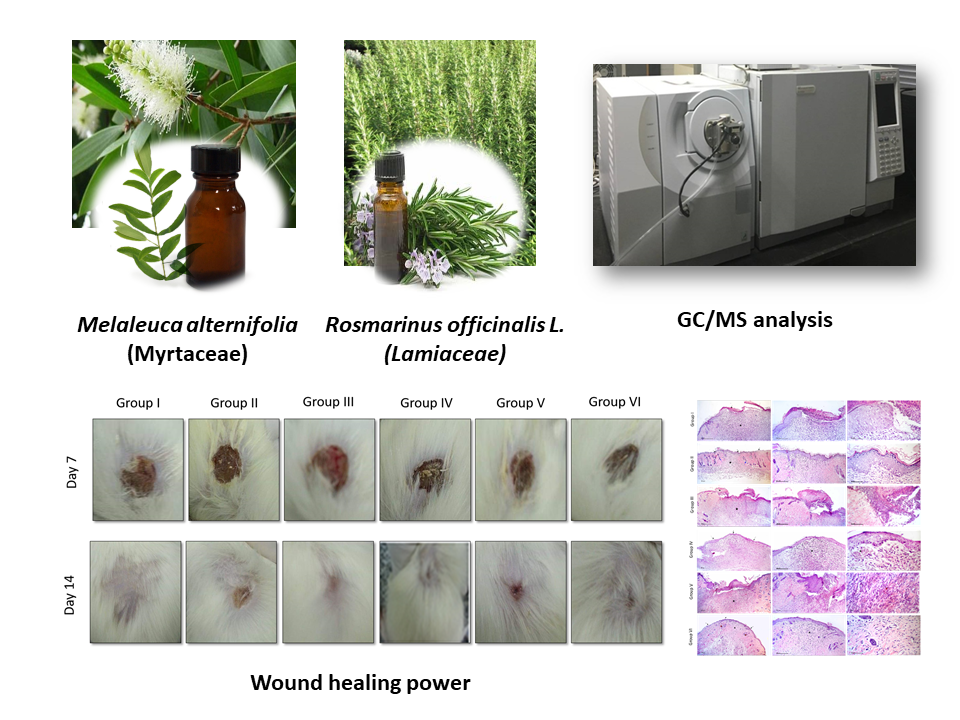

Supplement: S23 Fig — (TIF) [file pone.0219561.s023.TIF]
