# Supplementary material for: Appraisal on the wound healing potential of Melaleuca alternifolia and Rosmarinus officinalis L. essential oil-loaded chitosan topical preparations
Source: PLoS One. 2019 Sep 16;14(9):e0219561. doi: 10.1371/journal.pone.0219561 (PMC6746351; doi:10.1371/journal.pone.0219561)
Supplement: S22 Fig — (PDF) [file pone.0219561.s022.pdf]

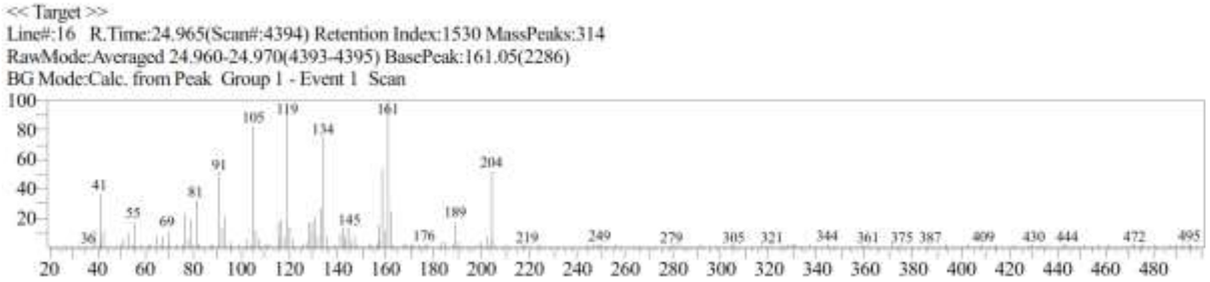

S22 Fig. EI/MS spectrum of compound (22) identified as δ-Cadinene in the essential oil of *M. alternifolia*
